# Verbal workplace violence in the health sector in Saudi Arabia: A cross-sectional study

**DOI:** 10.1097/MD.0000000000036760

**Published:** 2023-12-22

**Authors:** Reem Tarik Alsaqat, Aseel Khaled Alhassan, Fahad Saleh Al Sweleh

**Affiliations:** a Department of Dentistry, Restorative Division, Princess Nourah bint Abdul Rahman University, Riyadh, Saudi Arabia; b Department of Dentistry, King Khalid Hospital in AlKharj, Ministry of Health, Riyadh, Saudi Arabia; c Dental clinic, King Saud University, Riyadh, Saudi Arabia.

**Keywords:** healthcare workers, Saudi Arabia, verbal violence, workplace

## Abstract

Psychological abuse, such as verbal abuse, has received less attention than physical violence because of the manifested bodily harm caused by physical violence; however, verbal abuse has the highest percentage of violence worldwide. The consequences of verbal abuse in Saudi Arabia are similar to those in other countries. This study aims to determine the prevalence of verbal workplace violence in 12 months, the circumstances related to the event, and the consequences to both the attacker and the target person at all healthcare providers in the Kingdom of Saudi Arabia. This cross-sectional study included all healthcare providers registered with the Saudi Commission for Health Specialties who had worked for more than one year in the health sector in Saudi Arabia by May 2019. The researchers distributed questionnaires to the participants via email. Descriptive statistics were used to represent the basic properties of the data. Correlations between categorically measured variables were explored using the chi-square test for independence. Overall, 7398 healthcare workers (HCWs) voluntarily participated in the study. Overall, 49.1% encountered verbal abuse. Those who worked in the private sector and in shifts, particularly evening shifts, were significantly exposed to verbal abuse. Furthermore, pharmacists, followed by physicians, had the highest prevalence of workplace violence. Workplace verbal violence is highly prevalent, making it a major concern. Patients and their relatives are mostly the offenders of HCWs. Psychological ordeal, increased overwork, and reduced work capacity are the consequences of verbal abuse. Most victims do not report verbal violence, and this may result in an incorrect estimation of the problem. Therefore, encouragement to report verbal violence and additional research in Saudi Arabia are required.

## 1. Introduction

Violence, as the World Health Organization defines it, is “The intentional use of physical force or power, threatened or actual, against oneself, another person, or against a group or community that either results in or has a high likelihood of resulting in injury, death, psychological harm, maldevelopment, or deprivation.”^[1]^ It is a major risk factor in the health sector, and it may affect half of the healthcare workers (HCWs) worldwide.^[2]^ The number and severity of assaults have generally increased, and it is causing a traumatic effect on the victims.^[2,3]^ Any type of violence can threaten the well-being of HCWs, affecting the dignity of many people worldwide and causing emotional damage to the victims.^[4]^ Further, this can influence the institution to which they belong as they feel less satisfied by working, causing less productivity and inadequate quality of care to patients.^[5]^ Typically, physical violence receives more attention than verbal abuse because of the manifested bodily harm of physical abuse; however, psychological violence is not less harmful. Psychological abuse includes verbal abuse, bullying/mobbing, harassment, and threats, and it is defined as “Intentional use of power, including threat of physical force, against another person or group, that can result in harm to physical, mental, spiritual, moral or social development.”^[6]^ Previous studies on healthcare show that the rate of verbal abuse is 57.6% globally,^[7]^ 60% in Barbados,^[8]^ 72.4% in Turkey,^[9]^ 78.2% in Costa Rica,^[10]^ 30.7% in Saudi Arabia,^[11]^ and 76.2% in China.^[12]^ Verbal abuse does not occur only in healthcare centers; Duran et al’s study in Turkey shows that it is the most common type in the general community.^[13]^ Most studies show a relatively high percentage of verbal violence. However, differences are observed in the results of these studies because of the lack of a clear definition of verbal violence, which is owing to the diverse nature of the health sector and cultures in different countries where work violence occurs^[14]^ and the different methodologies used.^[11]^

Owing to verbal abuse, victims are psychologically impacted,^[15]^ stressed,^[5]^ and emotionally exhausted; further, it may result in less productivity and patient care,^[9]^ less satisfaction with work, and more caution when dealing with patients/clients.^[16]^

Some of the reasons for the high prevalence of verbal abuse are the under-acceptable level of safety climate, low training levels on how to deal with violent events,^[10]^ and a low percentage of those taking legal action against such behavior.^[17]^

Although prior studies have focused on workplace violence (WPV), to the best of our knowledge, none have covered the whole of Saudi Arabia and all specialties. One study was conducted only at King Fahad University Hospital in Al-Khobar City and only with nurses,^[11]^ and another study was conducted only in two hospitals in Abha City.^[18]^ Additionally, few studies have focused on the association between WPV and independent risk factors, such as hospital characteristics (i.e., clinical role and rank).

This study aims to determine the prevalence of verbal WPV over 12 months starting from May 2018 to May 2019, the circumstances related to the event, and the consequences for the attacker and target person at all healthcare facilities in Saudi Arabia, as well as to identify which group of healthcare providers is most susceptible. The study was conducted to raise awareness in the healthcare community about verbal violence and its effects on healthcare workers.

## 2. Methods

### 2.1. Data collection

This cross-sectional study included all healthcare providers who were registered with the Saudi Commission for Health Specialty (SCFHS) and had been working for more than 1 year in the healthcare sector (governmental or private) in Saudi Arabia by May 2019. Students, interns, employees of the administrative department, and providers who were not registered in the SCFHS or had less than one year of work experience were excluded. We received 7398 responses to our survey: 3792 male responses and 3606 female responses. Of the respondents, 402 were in the age range of 20 to 29 years, 3752 were 30 to 39 years, 2143 were 40 to 49 years, 882 were 50 to 59 years, and 219 were above 60 years. A convenience sampling technique was used; all eligible participants (304,002: physicians, pharmacists, nurses, midwives, health specialists, healthcare technicians, and ambulances) were invited to participate in the study.

Data were collected using a modified self-administered questionnaire developed by the Joint Program on Workplace Violence in the Health Sectors of the World Health Organization, International Labor Organization, International Council of Nurses, and Public Services International. The modifications to the questionnaire exclusively involved omitting some irrelevant questions (i.e., those that did not apply to Saudi Arabia). We used self-administered survey questionnaires because they are ideal for achieving wide geographic coverage of the target population and dealing with sensitive topics, and they are less resource-intensive than other data-collection methods. The survey questionnaires could be delivered electronically, which maximized the scalability and speed of data collection while reducing costs. The questionnaire was translated into Arabic for staff who were not fluent in English.

A pilot test was conducted to assess reliability and validity by distributing the questionnaires to five participants from the following specialties: medicine, dentistry, nursing, and pharmacy. These participants were Arabic and English speakers and had clinical experience in validating Arabic translations to avoid misunderstandings. These practitioners were excluded from the main study.

The questionnaire included questions related to the demographic data of the respondents, workplace characteristics, experience of violent events during the previous 12 months, risk factors contributing to WPV, personal opinions, perceptions, attitudes, experiences, and participants’ knowledge of WPV. The researchers distributed the questionnaires to the study sample via email. To increase the response rate, the researchers sent reminder emails to the participants after two weeks.

### 2.2. Statistical analysis

The data were analyzed using SPSS, version 22 (IBM Corp., Armonk, NY). Descriptive statistics (frequency and table) were used to describe the basic features of the data. Continuous variables were expressed as mean and standard deviation, whereas categorical variables were expressed as frequencies and percentages. The Kolmogorov–Smirnov statistical test of normality and histograms were used to assess the statistical normality assumption of the metric variables. The assumption of the statistical homogeneity of variance was evaluated using Levene’s test of homogeneity of variance. The chi-square test of independence was used to explore the correlations between the categorical variables. An independent samples *t* test was used to assess the mean differences in the continuous variables across the levels of categorically binary measured variables.

A multivariate binary logistic regression analysis was conducted to assess the combined and individual associations between the relevant predictors of the exposure of HCWs to recent physical violence at the workplace. The association between the measured predictor variables and their outcomes was expressed as odds ratios with a 95% confidence interval. Statistical significance was set at *P* < .05.

### 2.3. Ethical approval

This study was conducted according to the guidelines of the Declaration of Helsinki. Approval was obtained from the institutional review board of King Saud University College of Medicine (approval number: E-18-3391). Written informed consent for participation, publication, and confidentiality was obtained from the study participants at the beginning of the survey.

## 3. Results

### 3.1. Demographic characteristics

A total of 304,002 HCWs from the SCFHS database were included; only 7398 responded to the questionnaire. Among them, 51.3% were male and 48.7% were female; the mean age was 30 to 39 years (50.71%), and 60% were of non-Saudi origin. Nurses, midwives, and health specialists accounted for 38.1%; physicians for 30.91%; healthcare technicians and ambulance technicians for 25.54%; and pharmacists for 5.43% of the participants. Most of the participants were employed full-time (89.86%) in the public/governmental sectors (72.47%) (Table 1).

**Table 1:** Descriptive analysis of healthcare workers’ sociodemographic and professional characteristics (N = 7398).

Characteristics	n (%)
Sex	
Male	3792 (51.3)
Female	3606 (48.7)
Age	
20–29 years	402 (5.4)
30–39 years	3752 (50.7)
40–49 years	2143 (29)
50–59 years	882 (11.9)
≥ 60 years	219 (3)
Nationality	
Saudi	2957 (40)
Non-Saudi	4441 (60)
Clinical role	
Physicians	2287 (40)
Pharmacists	402 (5.4)
Nurses, midwives, and health specialist	2819 (38.1)
Healthcare technicians and ambulance	1890 (25.5)
Rank/seniority	
Junior	4605 (62.2)
Senior	1876 (25.4)
Consultant	917 (12.4)
Experience years	
1–5 years	851 (11.5)
6–10 years	2334 (31.5)
11–15 years	1905 (25.8)
16–20 years	1025 (13.9)
≥ 21 years	1283 (17.3)
Working sector	
Semi-governmental organization	380 (5.1)
Private sector	1656 (22.4)
Public/governmental sector	5362 (72.5)
Employment type	
Full-time	7256 (98)
Part-time	78 (1.1)
Temporary/casual	64 (0.9)

### 3.2. Experience of workplace violence

In the last 12 months, 49.1% of all respondents were exposed to some form of verbal abuse (verbal transgression or offense). It was observed that 37.1% of the verbal abusers were patients, and 36.7% were the relatives of patients. Further, 34.3% of the victims immediately asked the offender to stop their offense, whereas 27% reported the offense to their superiors, and 26.8% pretended it never happened (Table 2).

**Table 2:** Healthcare workers’ perceptions and experience of verbal workplace violence.

Variable	Total n (%)
Occurrence of verbal abuse in the last 12 months, n = 7398	
No	3763 (50.9)
Yes	3635 (49.1)
Typical incident of verbal abuse in workplace, n = 3312	
No	370 (11.2)
Yes	2942 (88.8)
The attacked person, n = 3312	
Patient/client	1230 (37.1)
Relatives of patients/client	1217 (36.74)
Staff member	261 (7.9)
Other	155 (4.7)
Management/supervisor	314 (4.2)
General public	70 (2.1)
External colleague/worker	65 (2)
Place of incident, n = 3312	
Inside health institution or facility	3178 (96)
Other place	76 (2.3)
At patient’s/client’s home	32 (1)
Outside (on way to work/ health visit/ home)	26 (0.8)
Response to the incident, n = 3312	
I told the offending person to stop	1136 (34.3)
I reported to senior staff member	901 (27.2)
Tried to pretend it never happened	887 (26.8)
took no action	856 (25.8)
I told a colleague	573 (17.3)
Completed incident/accident form	368 (11.1)
Told friends and/family members	299 (9)
Other	187 (5.6)
Transferred to another position	128 (3.9)
Sought counseling	122 (3.7)
Pursued prosecution	45 (1.4)
Sought help from the association	17 (0.5)
Sought help from the Saudi commission for health specialist	10 (0.3)
Completed a compensation claim	7 (0.2)
Preventability of incident, n = 3312	
No	1157 (34.9)
Yes	2155 (65.1)

### 3.3. Consequences of verbal violence

As shown in Table 3, 17.3% of those who were afflicted verbally believed an action was taken to investigate the event further by their superiors (85.8% were their managers). However, for 42.7% of those events, a verbal warning was issued to the offenders. The corrective and investigative actions taken to handle the verbal abuse event were between not satisfactory to slightly satisfactory on average (mean satisfaction = 2.17/5 satisfaction points). The primary reasons for not reporting verbal abuse were believing it was useless, fear of its negative consequences, and not knowing to whom to report the incident.

**Table 3:** Consequences of verbal violence.

Variable	Total n (%)
Bothering of attack	
a: Repeated, disturbing memories, thoughts, or images of the attack: mean (SD) Likert rating	2.97 (1.28)
b: Avoiding thinking or talking about the attack or avoiding having feelings related to it: mean (SD) Likert rating	3.10 (1.29)
c: Being “super-alert” or watchful and on guard: mean (SD) Likert rating	3.39 (1.30)
d: Feeling like everything you did was an effort: mean (SD) Likert rating	3.37 (1.27)
Investigating the causes of the incident, n = 3312	
Yes	572 (17.3)
No	2297 (69.4)
Don’t know	443 (13.4)
The person who takes action, n = 522	
Management/employer	448 (85.8)
Police	74 (14.2)
Other	44 (8.4)
Medical association	20 (3.8)
Saudi Commission for health specialists	16 (3.1)
Community group	12 (2.3)
Consequences to the attacker, n = 522	
Verbal warning issued	223 (42.7)
None	119 (22.8)
Don’t know	75 (14.4)
Reported to police	53 (10.2)
Other action	30 (5.7)
Care discontinued	17 (3.3)
Aggressor prosecuted	5 (1)
The offer of employer or supervisor, n = 1280	
Opportunity to speak about/report it	1062 (83)
Counselling	517 (40.4)
Other support	505 (39.5)
Incident handling satisfaction, n = 3167 Mean (SD) Likert rating, 1 = V. dissatisfied, 5 = V. satisfied,	
Very dissatisfied	1380 (43.56)
Dissatisfied	575 (18.2)
Neutral	736 (23.2)
Satisfied	251 (7.9)
Very satisfied	225 (7.1)
Reason for not reporting incident, n = 3167	
I thought it was useless	1739 (54.9)
I was afraid of negative consequences	890 (28.1)
It was not important	503 (15.9)
I did not know to whom to report the incident	400 (12.6)
Other reasons	318 (10)
I felt ashamed	168 (5.3)
I felt guilty	34 (1.1)

### 3.4. Experience of verbal attacks and their sociodemographic and professional factors

The findings showed that female HCWs were significantly less verbally abused than male HCWs (*P* < .001). Additionally, Saudi national HCWs were significantly more exposed to verbal abuse than non-Saudi workers, *P* < .001. Further, according to the chi-squared test of independence, physicians and pharmacists were significantly more verbally abused in the last year than nurses and technicians, *P* < .001. Senior HCWs were significantly more verbally abused on average than juniors and consultants, *P* < .001. However, the sector and job type of the HCWs did not converge significantly with their exposure to verbal abuse (Table 4).

**Table 4:** Association between healthcare workers’ experience of verbal abuse at the workplace and their sociodemographic and professional factors.

Variable	Verbally abused in your workplace (%), n = 7398			
	No = 3763	Yes = 3635	Test statistic	*P* value
Sex				
Male	1833 (48.7)	1959 (53.9)	χ^2^ (1) = 19.87	<.001
Female	1930 (51.3)	1676 (46.1)		
Age				
20–29 years	175 (4.7)	227 (6.2)	χ^2^ (4) = 140.84	<.001
30–39 years	1697 (45.1)	2055 (56.5)		
40–49 years	1203 (32)	940 (25.9)		
50–59 years	544 (14.5)	338 (9.3)		
≥60 years	144 (3.8)	75 (2.1)		
Nationality				
Saudi	1378 (36.6)	1579 (43.4)	χ^2^ (1) = 35.83	<.001
Non-Saudi	2385 (63.4)	2056 (56.6)		
Clinical role				
Physicians	1073 (28.5)	1214 (33.4)	χ^2^ (3) = 54.11	<.001
Pharmacists	154 (4.1)	248 (6.8)		
Nurses, midwives and health specialists	1505 (40)	1314 (36.1)		
Healthcare technicians and ambulance	1031 (27.4)	859 (23.6)		
Rank/seniority				
Junior	2436 (64.7)	2169 (59.7)	χ^2^ (2) = 25.04	<.001
Senior	864 (23)	1012 (27.8)		
Consultant	463 (12.3)	454 (12.5)		
Experience years				
1–5 years	421 (11.2)	430 (11.8)	χ^2^ (4) = 78.52	<.001
6–10 years	1057 (28.1)	1277 (35.1)		
11–15 years	955 (25.4)	950 (26.)		
16–20 years	561 (14.9)	464 (12.8)		
> 20 years	769 (20.4)	514 (14.1)		
Working sector				
Other-semi-governmental/private organization	185 (4.9)	195 (5.4)	χ^2^ (2) = 3.66	.160
Private – for profit sector	814 (21.6)	842 (23.2)		
Public/governmental sector	2764 (73.5)	2598 (71.5)		
Employment type				
Full-time	3689 (98)	3567 (98.1)	χ^2^ (2) = 2.25	.325
Part-time	45 (1.2)	33 (0.9)		
Temporary/casual	29 (0.8)	35 (1)		

### 3.5. Experience of verbal attacks and their working conditions

HCWs who worked in shifts, particularly those working in the evening (18:00–07:00), were significantly more exposed to verbal violence in the workplace (*P* < .001) (Table 5). Additionally, direct physical contact with the patients was a significant predictor of verbal abuse (*P* < .001). Further, those working with both patient sexes (male and female) were significantly more exposed to verbal offenses (*P* < .001).

**Table 5:** Association between healthcare workers’ experience of verbal abuse at the workplace and their working conditions.

Variable	Verbally abused at workplace (%), n = 7398			
	No = 3763	Yes = 3635	test statistic	*p*-value
Work in shifts				
No	1779 (47.3)	1402 (38.6)	χ^2^ (1) = 57.97	<.001
Yes	1984 (52.7)	2233 (61.4)		
Working time between 18:00 (6 pm) and 07:00 (7 am)				
No	1553 (41.3)	1168 (32.1)	χ^2^ (1) = 66.40	<.001
Yes	2210 (58.7)	2467 (67.9)		
Interacting with patients/clients				
No	565 (15)	182 (5)	χ^2^ (1) = 204	<.001
Yes	3198 (85)	3453 (95)		
Routine direct physical contact (washing, turning, lifting) with patients/clients				
No	1659 (44.1)	1726 (47.5)	χ^2^ (2) = 203.7	<.001
Yes	1542 (41)	1727 (47.5)		
Not applicable	562 (14.9)	182 (5)		
Patients/clients you most frequently work with (tick all appropriate)				
Newborns	655 (17.4)	715 (19.7)	χ^2^ (1) = 6.27	.012
Infants	755 (20.1)	858 (23.6)	χ^2^ (1) = 13.60	<.001
Children	1263 (33.6)	1488 (40.9)	χ^2^ (1) = 43.02	<.001
Adolescents	1541 (41)	2006 (55.2)	χ^2^ (1) = 150.10	<.001
Adults	2755 (73.2)	3084 (84.4)	χ^2^ (1) = 150.33	<.001
Elderly	1862 (49.5)	2364 (65)	χ^2^ (1) = 182.60	<.001
Sex of the patients you most frequently work with				
Unspecified/NA	562 (14.9)	182 (5)	χ^2^ (3) = 210	<.001
Female	294 (7.8)	279 (7.7)		
Male	339 (9)	311 (8.6)		
Male and female	2568 (68.2)	2863 (78.8)		

### 3.6. Experience of verbal attacks and characteristics from hospital violence reporting guidelines

Violence-related worry was more common among survivors (mean score, 3.34/5 points using a Likert scale; standard deviation (SD) = 1.2) than among those who were not exposed (mean score, 2.36, SD = 1.26) (*P* < .001). Additionally, HCWs working in facilities with dedicated procedures and guidelines for reporting and managing work violence events had relatively low rates of exposure to verbal violence (*P* < .001) (Table 6). Further, encouragement from facility administration to report violence resulted in significantly less exposure to verbal offenses than those in workplaces without violence intolerance. In particular, the encouragement of violence reporting from managers and colleagues, as well as the SCFHS and medical associations, resulted in significantly less exposure to verbal offenses at the workplace (*P* < .050).

**Table 6:** Association between healthcare workers’ experience of verbal abuse at the workplace and their hospital violence reporting guidelines characteristics.

Variable	Verbally abused in your workplace (%), n = 7398			
	No = 3763	Yes = 3635	Test statistic	*P* value
Worried about violence in the current workplace: Mean (SD)	2.36 (1.26)	3.34 (1.20)	t (7396) = 33.95	<.001
Presence of procedures for reporting of violence				
No	950 (25.2)	1146 (31.5)	χ^2^ (1) = 35.90	<.001
Yes	2813 (74.8)	2489 (68.5)		
Knowing how to use the report				
No	375 (13.3)	408 (16.4)	χ^2^ (1) = 9.80	.002
Yes	2438 (86.7)	2081 (83.6)		
Encouragement to report workplace violence				
No	1003 (26.7)	1736 (47.8)	χ^2^ (1) = 353.17	<.001
Yes	2760 (73.3)	1899 (52.2)		
Person who encourages reporting				
Management/ employer/	2307 (61.3)	1625 (44.7)	χ^2^ (1) = 204.7	<.001
Colleagues	803 (21.3)	681 (18.7)	χ^2^ (1) = 7.8	.005
Saudi commission for health specialist	342 (9.1)	228 (6.3)	χ^2^ (1) = 20.61	.003
Medical association	109 (2.9)	72 (2)	χ^2^ (1) = 6.50	.011
My own family/ friends	139 (5.1)	183 (5)	χ^2^ (1) = 0.03	.853
Other persons	165 (4.4)	142 (3.9)	χ^2^ (1) = 1.1	.302

### 3.7. Multivariate logistic binary regression analysis results

**Figure 1.**
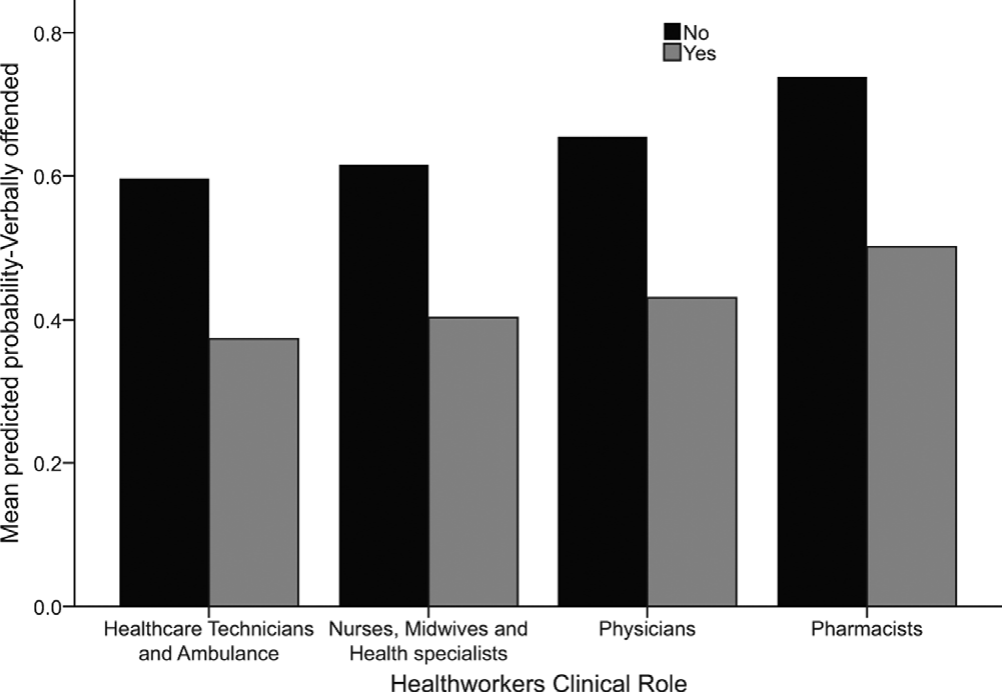
Difference in the probability of being verbally abused according to clinical roles and encouragement to report violence in their workplace.

There were no statistically significant differences between male and female HCWs regarding exposure to verbal intimidation/abuse in the workplace in the last year (*P* = .711). HCWs aged between 20 and 29 years were significantly more (43.9% times more) verbally abused than those aged 40 years and older (*P* < .001). Those aged 30 to 39 years had significantly greater (36.5% times higher) exposure to verbal offense at the workplace than those aged 40 years or older (*P = *.003). However, the HCWs’ seniority level at work did not converge significantly on their possibility of being verbally abused (*P* = .146). Pharmacists were more verbally abused (76 times higher) at the workplace than nurses and technicians (*P* < .001); likewise, physicians were more verbally offended at the workplace (32.5% times more) than nurses and medical technologists on average (*P* = .001) (Fig. 1). The HCWs working in direct physical contact with the patients had slightly greater, though not statistically significant, odds of verbal offense exposure than those not working in direct physical contact (*P* = .203). Further, those working evening shifts were associated with slightly high odds of (12% times higher) verbal abuse exposure (*P* = .052). HCWs with daily interactions with their clients and patients had significantly higher odds of verbal offenses than those without daily interactions with patients while working (*P* < .001) (Table 7).

**Table 7:** Multivariate logistic binary regression analysis of the predictors of healthcare workers’ exposure to verbal abuse at the workplace (N = 7398).

	Multivariate adjusted odds ratio (OR)	95% CI for OR		*P* value
		Lower	Upper	
Interacting daily with patients	2.239	1.697	2.955	<.001
Job = Pharmacists	1.760	1.367	2.265	<.001
Worry level from violence at work: mean score	1.690	1.621	1.762	<.001
Encouragement for reporting by significant other persons	1.630	1.246	2.131	<.001
Encouragement for reporting by direct managers	1.490	1.237	1.794	<.001
Age: 20–29 years	1.439	1.129	1.834	.003
Age: 30–39 years	1.365	1.216	1.533	<.001
Job = Physicians	1.325	1.124	1.561	.001
Encouragement for reporting by colleagues	1.287	1.119	1.480	<.001
Working with elderly patients	1.254	1.103	1.426	.001
Work sector = Semi-governmental	1.187	0.942	1.495	.146
Work sector = Private sector	1.154	1.008	1.320	.038
Working with adolescent patients	1.134	1.002	1.283	.046
Working in evening shifts	1.118	0.999	1.251	.052
Presence of violence reporting guidelines in the workplace	1.122	0.985	1.279	.084
Working in evening shifts	1.118	0.999	1.251	.052
Has direct physical contact with the patients	1.078	0.9603	1.210	.203
Seniority/Rank level	1.048	0.949	1.157	.358
Sex = Female	0.979	0.874	1.096	.711
Nationality = None Saudi	0.835	0.741	0.943	.003
Encouragement for reporting by the Saudi Commission	0.828	0.679	1.011	.064
Reporting of violence encouraging workplace	0.333	0.270	0.412	<.001
Constant	0.097			<.001

### 3.8. The differences in the probability of being verbally abused, according to clinical roles and encouragement to report violence in their workplace

Figure 1 shows that pharmacists have greater odds of being abused verbally (76 times higher) at the workplace than nurses and technicians. Further, physicians were at greater risk of being verbally offended in the workplace (32.5% times more) than nurses and medical technologists on average. Additionally, the presence of a violence reporting procedure and guidelines did not significantly converge on the odds of HCWs’ exposure to verbal offense at work. However, the presence of such guidelines predicted slightly statistically high exposure to verbal abuse. More interestingly, even though the presence of procedures and guidelines did not converge significantly on the HCWs’ odds of verbal offense, the encouragement of the facility administration to report violence predicted significantly low odds (66.7% times less) of verbal offense experiences.

## 4. Discussion

This study aimed to determine the prevalence of verbal WPV, the circumstances related to the event, and the consequences for both the attacker and the target person among all healthcare providers in the KSA.

The disfiguration and lack of clarity in the definition of WPV led to increased violence. In general, the abuse of HCWs accounts for approximately a quarter of all work violence events; some of these situations have just been observed, and some are the norm to some HCWs.^[19]^ The most interesting finding of our study is that verbal abuse was the most common type of violence experienced by HCWs, as shown in other studies.^[6,11,20]^

This study examined verbal abuse against healthcare providers in all the cities in the KSA. According to our study, 49.1% of the participants had experienced verbal abuse in the last 12 months. Conversely, Alsaleem et al, who studied nurses working at King Fahad University Hospital in Khobar, and Alshamlaan et al, who studied all HCWs in Abha,^[11,21]^ reported more exposure to verbal abuse than the current study. However, both studies were conducted in one hospital and cannot be generalized to all HCWs in the Kingdom. Additionally, most international studies^[22–25]^ reported higher results than this study.^[22–25]^ The main reasons for the universal exposure to verbal violence are the lack of education, patient cultural background and personality, workload, social and economic situation,^[6,21]^ emotional manifestation of pain in psychiatric disorders, and alcohol and other substance abuse, which may influence people’s behavior.^[19]^ Forrest et al found that working full-time or in large hospitals was associated with an increased prevalence of verbal abuse. However, compared with this study, job type was not significant. Additionally, those who had fewer years in practice were more likely to experience verbal abuse than those with more experience; in the present study, those who had 6 to 10 years of experience had the highest exposure to verbal violence.^[26]^

In this study, according to the HCWs, the offending persons were mostly the patients and their relatives. This is consistent with the findings of Abed et al^[8]^ and some other studies.^[5,10,27–29]^ In contrast, in the study by Samir et al, half of the abuses came from colleagues and staff members.^[20]^ According to the present study, the working sector and job type of HCWs did not significantly converge on their exposure to verbal abuse. However, another study in Europe shows that governmental hospitals are more vulnerable to verbal abuse than private hospitals.^[30]^ Social problems related to psychological violence are one of the most common causes of verbal abuse, as presented in the study in Turkey by Kisa et al, which mentioned that misunderstandings and personal problems of perpetrators are the reasons for verbal WPV.^[9]^ Saudi Arabia has the same issue since many non-Saudi healthcare practitioners are working in the country, and differences in languages, cultures, and religions lead to miscommunications.^[31]^

Verbal violence could worsen the mental and emotional status of HCWs and could cause them to be “super-alert.” The study by Kisa et al^[9]^ showed that anger was the most observed emotional reaction to verbal abuse, followed by shock/surprise. Many studies have reported experiences of violent incidents resulting in severe psychological distress, increased work stress, and reduced work efficiency.^[32,33]^ In this study, we observed that verbal warning was the most common action taken against verbal abuse, followed by no action, and the least frequent reaction was aggressor prosecution. In contrast, in a study by Li et al,^[34]^ no action was found to be the most common, as reported by general practitioners and nurses, followed by a verbal warning issued by the hospital manager; the least action was reporting it to the police. This study is aligned with ours in that many healthcare practitioners do not know how to report the incident, even if there is an existing reporting system in their workplace.

In response to psychological violence, most of the practitioners in this study told the offending person to stop or reported it to a senior staff member. Conversely, in Li et al’s study in a Chinese hospital,^[34]^ most general practitioners and nurses pretended that nothing happened. Our study showed that the incidents were not reported because the victims thought it was useless and were afraid of negative consequences, which is consistent with the previous report,^[34]^ as most victims only reported incidents resulting in physical injuries.

The participation of HCWs in the governmental and private sectors in Saudi Arabia strengthened our study compared with other studies that focused on one department or one clinical role.

## 5. Conclusions

In this study, we found that verbal abuse was the most common form of violence experienced by HCWs in Saudi Arabia, affecting approximately half of the HCWs. However, those working night shifts and in private sectors, pharmacists, and physicians had a higher chance of being verbally abused than nurses, technicians, and medical technologists. Most victims did not report verbal violence, which led to the incorrect estimation of the problem. Creating a comfortable and safe environment for HCWs is a crucial management policy. Therefore, additional research in Saudi Arabia is needed to clarify the definition of verbal abuse or violence in educational processes.

The limitations of this study include its retrospective design and the use of self-reported questionnaires, which might have introduced some recall bias. Further, verbal abuse and violence were not clearly defined. The participants were unsure about their exposure to verbal abuse or violence owing to the lack of exact definitions; thus, judgments might have been vague. Further studies are needed to understand the different reasons for this type of violence, which will help provide suitable solutions.

## Acknowledgments

A special thanks to the Saudi Commission for Health Specialties who helped us reach healthcare providers registered at their institutions.

## Author contributions

**Conceptualization:** Fahad Saleh Al Sweleh.

**Data curation:** Reem Tarik Alsaqat, Aseel Khaled Alhassan, Fahad Saleh Al Sweleh.

**Formal analysis:** Reem Tarik Alsaqat, Aseel Khaled Alhassan, Fahad Saleh Al Sweleh.

**Investigation:** Reem Tarik Alsaqat, Aseel Khaled Alhassan.

**Methodology:** Reem Tarik Alsaqat, Aseel Khaled Alhassan, Fahad Saleh Al Sweleh.

**Project administration:** Reem Tarik Alsaqat, Fahad Saleh Al Sweleh.

**Resources:** Reem Tarik Alsaqat, Aseel Khaled Alhassan, Fahad Saleh Al Sweleh.

**Software:** Reem Tarik Alsaqat, Aseel Khaled Alhassan, Fahad Saleh Al Sweleh.

**Supervision:** Reem Tarik Alsaqat, Fahad Saleh Al Sweleh.

**Validation:** Reem Tarik Alsaqat, Aseel Khaled Alhassan, Fahad Saleh Al Sweleh.

**Visualization:** Reem Tarik Alsaqat, Fahad Saleh Al Sweleh.

**Writing – original draft:** Reem Tarik Alsaqat, Aseel Khaled Alhassan, Fahad Saleh Al Sweleh.

**Writing – review & editing:** Reem Tarik Alsaqat, Aseel Khaled Alhassan, Fahad Saleh Al Sweleh.
